# Two implant overdenture–the first alternative treatment for patients with complete edentulous mandible

**Published:** 2011-05-25

**Authors:** M Melescanu Imre, M Marin, E Preoteasa, AM Tancu, CT Preoteasa

**Affiliations:** ‘Carol Davila’ University of Medicine and Pharmacy, Faculty of Dentistry, BucharestRomania

**Keywords:** edentulism, quality of life, implantology

## Abstract

Given the increasing life expectancy in the coming years, dental practitioners, as other specialists from different medical fields, will encounter an increasing number of complete edentulous patients. These patients, with a longer active life and higher standards of life quality, will have different expectations for their complete dentures, higher than the standard treatment that uses conventional complete dentures.

Two–implant overdenture is considered the first alternative treatment in complete edentulous mandible, according to current medical standards established by a team of specialists in prosthodontics and implantology, and globally known as the McGill Consensus from McGill University, Montreal, Canada. The Consensus was established during a–dayߝand–a–half session of presentations done by experts who presented data, scientific information on the subject, and, not less significant, personal experiences of participants and patients. Overdenture on implants, as an alternative treatment for complete edentulous mandible, has multiple benefits in achieving better conditions of prosthesis: balance and effectiveness, with positive effects on oral structures, aesthetics, and quality of life. Mandibular two–implant overdenture, established as a standard treatment by the highest international forum, should gradually become the first choice of treatment in complete edentulous mandible.

## Introduction

In the current practice of dentistry, more implant treatment is increasingly required. Today, in 70% of periodontal prosthesis cases worldwide, an implant is preferred. The same tendency is observed in complete edentulous patients, as the complex pathology is associated with multiple physiological aging phenomena and with all clinical aspects of geriatric patient. This paper provides an update of the treatment methods in accordance with the new standards of life of complete edentulous patients.

Given the increasing number of complete edentulous patients worldwide, and also because of some patients' complaints regarding the conventional dentures, especially related to the lower jaw, the experience of both professionals and patients has shown the need to establish clear principles of treatment with implants overdenture such as the number and locations of implants, and the type of implants loading. In this respect, McGill University adopted, in 2002, a Consensus meant to draw directions for this type of treatment that will became very popular because of the many advantages it brings to patients, significantly improving their quality of life. 

### McGill Consensus–Definition

McGill Consensus refers to the newly accepted standard of care procedure of two–implant overdenture, placed interforaminal, with the possibility of immediate loading, as the primary treatment for complete mandibular edentulous patients.

At McGill University–Canada, a University with an old tradition in the applicability of clinical research and practice of multiple prosthetic systems, a Consensus regarding the best modality for the treatment of the complete edentulous mandible was discussed and adopted in May 2002. 

The Consensus has been debated for two days and adopted in a Symposium. Experts, having clinical experience in the field and knowledge from the researched literature attended the event and expressed their opinions. The team of specialists, who have formed the Consensus, comprised the most prestigious names in the world of prosthodontists–J.S. Feine, DDS, MS, HDR, CANADA and G. E. Carlsson, LDS to appoint only two of the participants. The conclusions of the studies have been analyzed in order to determine the current treatment standards for complete edentulous mandible and to establish new standards of treatment. [1]

According to the specialists, complete edentulous patients, whose experiences were discussed as a part of the study group, were invited to participate at the Consensus.

**Figure 1:**
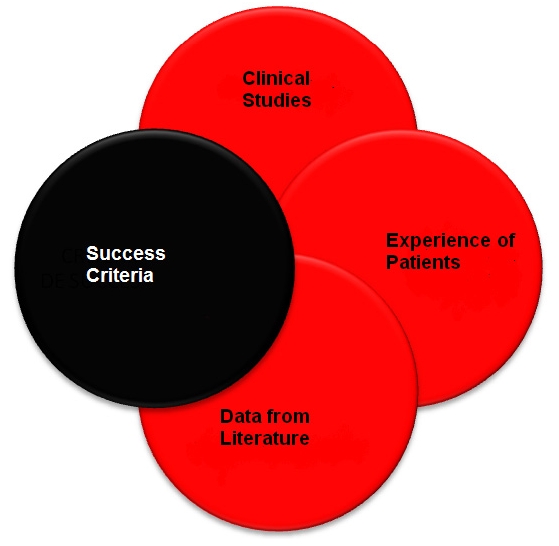
Criteria considered in establishing the Consensus

Therefore, based on numerous clinical and in vitro studies, the two–implants overdenture as treatment for complete edentulous mandible was established to be the main treatment, superior to the conventional complete dentures, because of the real benefits it brings to the patient. The Consensus was adopted by the vast majority of specialists. [2] 

### The Conclusions of the Consensus 

Please note that this work is an independent study, strictly based on scientific and therapeutic research without any commercial or political implications. 

Based on studies conducted on a large sample of patients, globally, it was shown that the total number of edentulous persons is growing in all industrialized countries, and will continue to grow because of the increase of life expectancy. [3] In the same time, the expectations of the patients and their life style require a superior level of convenience and functionality compared with what the conventional complete denture can offer.

For more than 100 years, maxillary and mandibular complete dentures have been the standard treatments for complete edentulous patients. If the majority of patients can tolerate the maxillary prosthesis, the situation is different for the mandibular prosthesis. The instability and discomfort it causes to patients represents the starting point in establishing the two–implant overdenture as the primary alternative treatment for patients with complete edentulous mandible. 

Regarding the applicability of implantology, it is well known through the numerous publications on longitudinal studies [4] that the success rate of implants placed in the anterior mandible is very high and with minimal clinical impediments. In addition, the positive effect of implants on the mandibular ridge resorption has also been scientifically proven. [5,6] 

Regarding the patient's satisfaction, most patients with conventional mandibular denture accused masticatory disturbances and changes in diet, leading to nutritional deficiencies. [7] The superiority of the overdenture on implants (regardless of the type of attachment system used) in comparison with conventional complete dentures has been demonstrated by numerous studies measuring patient satisfaction. It demonstrated a major improvement in mastication, phonation, and denture stability. Two–implant overdenture also confirmed a significant improvement in the patients' quality of life with positive results on edentulous patients' general health and, consequently, reducing their total healthcare costs in the long term [8]. 

In terms of immediate costs of two–implant overdenture, it is obvious that they are higher than the conventional complete denture. According to the literature data, the authors recommend the insertion of the implants at the same time, to reduce the surgery costs for the patient. [9].

However, being acquainted with the main benefits brought by this type of treatment, the authors concluded that the two–implant overdenture should be applied as the primary treatment option in complete edentulous mandible patients, while conventional complete denture should be considered an emergency treatment. 

Many prestigious publications–‘Gerodontology’, ‘Quintessence’, ‘International Journal of Prosthodontics’, ‘European of Prosthodontics and Restorative Dentistry Journal’–like the majority of the most renowned academic publications–agreed with the authors and adopted ‘The Consensus of McGill’ in specialty publications and in clinical environment, establishing the two–implant mandibular overdenture as the ‘standard of care’ for therapy in complete edentulous mandible patients.

The prosthesis on implants has numerous advantages such as better balance, increased functional efficiency, safer mastication, diminished ridge resorption, improved aesthetics, and especially eliminates the fear of detachment in speech or mastication (unpleasant aspects, particularly in situations when patients are in the company of others). The implants are strong, durable, and prevent a number of oral modifications and prosthetic shortcomings. 

It should, however, be indicated that the implant overdenture treatment is more complex than the conventional prosthesis, and requires at least two distinct phases: surgical and prosthetic, both of them having risks, high costs, and consequences on the final outcome of the treatment. 

Therefore, based on similar principles, some authors have proposed the use of mini implants which have lower risks and costs, but similar end results. [10, 11]

Overdenture on implants, is an alternative to conventional denture, with undeniable benefits, but it is not a risk–free process. Although patient selection criteria are clearly stated in terms of medical practice, they must be adapted for each particular clinical situation, the practice often providing a challenge, and the selection being made based on non–medical criteria. The dental practitioner may also struggle with myths and false beliefs. Last but not least, the responsibility and reputation of the dental practitioner whose career is built as a ‘domino’ is important, any failure having negative consequences on the ‘domino construction’ and every success being a new piece that builds the ‘domino’. [12] 

## Conclusions

In conclusion, the mandibular two–implant overdenture placed interforaminal, with the possibility of immediate load, adopted by the high forum of the Consensus’ professionals, and supported by the vast majority of dental practitioners around the world, must be the first choice of treatment, recognized as the standard treatment for patients with complete edentulous mandible. The mandibular two–implant overdenture has important medical and bioethical implications, and significant impact on short and long term costs. The alternative of mandibular two–implant overdenture has better benefits and lower costs than the fixed prostheses on implants. It is preferred when considering the clinical typology of complete edentulous patients, mostly associated with illnesses that prevent multiple and laborious surgeries, such as those imposed by the insertion of at least six implants, like the case of mandibular fixed prosthesis. Mandibular two–implant overdenture must gradually become the first treatment option in complete edentulous mandible patients. 
